# Simultaneous imaging of ultrasonic relative backscatter and attenuation coefficients for quantitative liver steatosis assessment

**DOI:** 10.1038/s41598-023-33964-9

**Published:** 2023-06-01

**Authors:** José Timaná, Hector Chahuara, Lokesh Basavarajappa, Adrian Basarab, Kenneth Hoyt, Roberto Lavarello

**Affiliations:** 1 Laboratorio de Imágenes Médicas, Pontificia Universidad Católica del Perú, Lima, Peru; 2 Department of Bioengineering, University of Texas at Dallas, Richardson, TX USA; 3 INSA-Lyon, UCBL, CNRS, Inserm, CREATIS UMR 5220 U1294, Université de Lyon, Villeurbanne, France

**Keywords:** Biomedical engineering, Acoustics

## Abstract

Prevalence of liver disease is continuously increasing and nonalcoholic fatty liver disease (NAFLD) is the most common etiology. We present an approach to detect the progression of liver steatosis based on quantitative ultrasound (QUS) imaging. This study was performed on a group of 55 rats that were subjected to a control or methionine and choline deficient (MCD) diet known to induce NAFLD. Ultrasound (US) measurements were performed at 2 and 6 weeks. Thereafter, animals were humanely euthanized and livers excised for histological analysis. Relative backscatter and attenuation coefficients were simultaneously estimated from the US data and envelope signal-to-noise ratio was calculated to train a regression model for: (1) fat fraction percentage estimation and (2) performing classification according to Brunt’s criteria in grades (0 <5%; 1, 5–33%; 2, >33–66%; 3, >66%) of liver steatosis. The trained regression model achieved an $R^2$ of 0.97 (*p*-value < 0.01) and a RMSE of 3.64. Moreover, the classification task reached an accuracy of 94.55%. Our results suggest that *in vivo* QUS is a promising noninvasive imaging modality for the early assessment of NAFLD.

## Introduction

Nonalcoholic fatty liver disease (NAFLD) has become one of the most common etiologies of chronic liver disease in the world. It is a metabolic condition characterized by a complex interplay of hormonal, dietary, and hereditary variables in which excess fat is stored in your liver that includes a spectrum ranging from simple steatosis, steatohepatitis (NASH) with or without different stages of fibrosis, cirrhosis, and hepatocellular carcinoma (HCC)^1^. The global prevalence of NAFLD is on rise in both developed and developing nations. Among adults, it is estimated to be between 20-25%, with the Middle East (32%) and South America (30%) having the greatest prevalence, followed by Asia, USA, and Europe, and the lowest in Africa (13%), also it is estimated that 20% of people with NAFLD are affected by NASH^2,3^. As a result, NAFLD has become the most common cause of chronic liver disease around the world^4^.

The current gold standard for diagnosing NAFLD is a needle biopsy procedure. In the last decades, attempts were made to develop standardized histological evaluation systems, including the NAFLD activity score (NAS)^5^, and steatosis, activity and fibrosis (SAF) score^6,7^. However, even with the use of standardized metrics, one of the major limitations is the high rate of sampling error^8^ which is usually inevitable, due to the fact that most parenchymal abnormalities are unevenly distributed in virtually all liver disorders. Moreover, since liver biopsies sample only $1/50,000^{th}$ of the total liver mass, the small piece of tissue that is actually analyzed may be insufficient to generate an appropriate diagnosis^9–11^. Reports suggest that due to this sampling problem, liver biopsy can fail to detect nonalcoholic steatohepatitis (NASH) in up to 24% of cases and unsuccessfully determine the level of fibrosis in 22 to 37% of cases^12^. Notwithstanding, it is an invasive method, highly dependent on the operator’s experience, that generates stress, discomfort, and additional costs to the patient, and could even cause clinical complications in about 1 in 200 patients^13^.

Laboratory tests (serological methods) are an alternative tool to diagnose NAFLD, but have known limitations. The first one is that normal values of some liver tests like alanine aminotransferase (ALT) levels have been shown to cover the full spectrum of NAFLD^14^. Overall, the use of steatosis biomarkers has performed modestly in studies of hundreds of patients^15^. While there are serological panels that have been developed for the diagnosis of hepatic steatosis (e.g., SteatoTest)^16^, performance has produced area under the curve values between 0.7 and 0.8^17,18^.

Another alternative to liver biopsy is the use of medical imaging. Conventional ultrasound (i.e., ultrasound, US) is one of the preferred radiological modalities for the study of NAFLD^19^ because of its safety, little associated patient discomfort, availability and lower instrumentation costs, which provides multiple advantages over more sensitive techniques such as magnetic resonance imaging derived proton-density-fat-fraction (MRI-PDFF)^20^. Particularly, NAFLD diagnosis by US is based on the presence of (1) hyperechogenicity in the liver parenchyma that can be quantified by the hepatorenal index (HRI)^21^, and (2) the rise in US attenuation in liver parenchyma with the subsequent reduction of the visualization of intrahepatic blood vessels^22^. Both of these characteristics are used in scoring systems such as the Hamaguchi score^23^. Nonetheless, this technique has several deficiencies in the diagnosis of NAFLD. First, US does not allow quantification of the degree of steatosis or fibrosis, it is mainly a qualitative technique that depends on radiologist’s skill and experience to locate abnormal structures by visual inspection and differential comparison. Second, the effectiveness of US for the diagnosis of NAFLD can be dramatically reduced in morbidly obese patients with sensitivities below 40%^24^. Third, US requires a degree of hepatic infiltration greater than 30% for the proper detection of steatosis^25,26^.

The lack of quantitative information leads to increased follow-ups or invasive biopsies that could otherwise be unnecessary given the unused quantitative information available in medical images^27^. Quantitative US (QUS) imaging techniques have the potential to solve this problem with minor interoperator variability^28^. They are used to characterize tissue microstructure and to infer the acoustical properties of the tissue. Spectral-based QUS parameters, such as the attenuation coefficient (AC, expressed in dB/cm/MHz) and the backscatter coefficient (BSC, expressed in $cm^{-1}\cdot sr^{-1}$), can be estimated directly from backscattered US signals. The AC is a measure of US energy loss in tissue and provides a numerical parameter analogous to the obscuration of tissue structures assessed qualitatively from B-mode US images. Conversely, BSC is a measure of US energy returned from tissue and provides a quantitative parameter analogous to the echogenicity assessed qualitatively from B-mode US images^29^. Both can be used to differentiate between normal and fatty livers^30^. The repeatability and reproducibility of AC and BSC techniques have similarly been tested and proven to be excellent interobserver and interplatform^31^. Therefore, AC and BSC are sensitive to accumulation of fat within the liver. Moreover, in contrast to histogram-based methods that analyze the information related to speckle patterns, AC and BSC are not affected by scanner settings such as gain and contrast, they are independent from diffraction effects and transducer transfer function, but require a reference phantom to be computed^32–34^.

Several studies have been performed to measure the progression of steaotosis using QUS techniques by estimating AC and BSC independently^35^. A study performed in 35 volunteers^36^, showed a significant difference in attenuation and BSC at 3MHz, mean BSC increased around 10 times its value and mean attenuation in fatty livers was 0.88 dB/cm higher. Ghoshal et. al.^30^ summarizes a group of studies performed in humans, in which attenuation increased between 0.5 dB/cm to 1 dB/cm at 3MHz in fatty livers with respect to normal livers across the studies. In the case of BSC, it increased approximately 10 times. In murine animal models^30^, demonstrates that similar trends can be found even if the frequency bandwidth of analysis is higher, thus showing that QUS parameters are correlated with the grade of fatty liver by comparing them with total liver lipids. As a point of reference, from male New Zealand white rabbits, AC values for the normal rabbit liver group with 15±9 mg lipids/g of liver were 0.71±0.05 dB/cm/MHz, while the fatty rabbit group liver with 139±28 mg lipids/g of liver obtained 1.27±0.02 dB/cm/MHz. In addition, Baek et. al.^37^ showed the importance of frequency-dependent attenuation correction at greater depths that may suffer from poor signal-to-noise conditions and its great contribution for discriminating between different stages of steatosis using multiparametric clustering analyses based on B-mode imaging.

The evidence suggests that, if both AC and BSC could be assessed simultaneously, diagnosis and classification of hepatic steatosis in NAFLD could be improved. Currently, there are already studies using QUS techniques to study hepatic steatosis, and the first commercial products are appearing on quantitative scans^29^. However, to decrease shadowing artefacts or determine scatterer concentration and statistical characteristics of the backscatter, a thorough understanding of tissue attenuation is essential^38^. That is why, in order to accurately estimate BSC, total attenuation should be compensated. For this purpose, three approaches have been described: manual compensation by individual tissue identification, compensation based on a single AC for each frequency, and, finally, parametric modeling, that would allow the simultaneous estimation of both total attenuation and BSC^39^. However, parametric modelling estimations are limited by the trade-off between the variance of the estimates and the spatial resolution^40–44^. To address this limitation, a framework for QUS parameters estimation was recently introduced^44^. Termed the regularized power law by total variation (RPL-TV), this low computational cost method was shown to overcome the accuracy-resolution tradeoff during QUS parameter estimation. Nevertheless, there is a latent need to validate feasibility of using simultaneous *in vivo* estimation methods for steatosis monitoring, a scenario in which the assumption of homogeneity in the region of analysis is not met due to the presence of heterogeneous tissue across the skin, fat layers, muscles, blood vessels, and other artifacts^45^.

## Results

**Figure 1:**
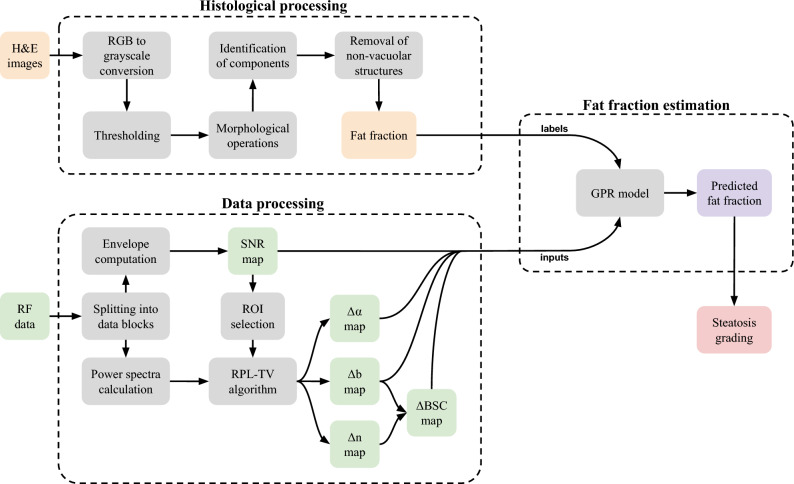
Flowchart with the steps taken to develop the proposed method.

We conducted experiments to validate our QUS technique in a preclinical setting with a group of 55 Sprague-Dawley divided into control and methionine and choline deficient (MCD) diet groups subjected to different diets to model liver steatosis. The RPL-TV framework was the algorithm selected to perform BSC and AC simultaneous estimation^46^. Estimated relative coefficient maps ($\Delta \text {b}$ and $\Delta \alpha$), relative BSC and envelope signal-to-noise ratio (SNR) median values in the region of interest were used to train the regression model. A flowchart describing the steps to develop the proposed method is shown in Fig. 1.

QUS imaging was performed to characterize liver tissue microstructure by measuring fundamental acoustical parameters. The goal of this research was to propose a method for quantifying the progression of hepatic steatosis based on quantitative ultrasound in a murine animal model. All procedures were carried out in accordance with relevant guidelines and regulations. All methods are reported in accordance with ARRIVE guidelines^47^. The RPL-TV framework was computed using volumetric radiofrequency (RF) data in a bandwidth of 4-18 MHz. Power spectrum is calculated on overlapped data blocks along each frame, which are also used for SNR map calculation. After the final imaging session at 2 weeks and 6 weeks, all animals were humanely euthanized and livers excised for histological processing. Binarized histological analysis of hematoxylin and eosin (H &E) images segmented diffuse fat deposits were used for fat fraction calculation.

**Figure 2:**
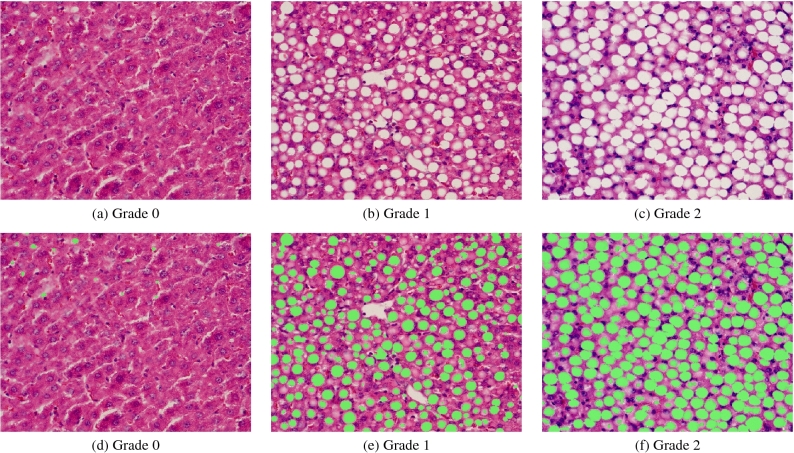
Representative stained liver tissue: (**a**)–(**c**) original H &E histology images, (**d**)–(**f**) processed H &E histology images with fat vacuoles highlighted in green.

Hepatic steatosis can be classified from grades 0-3^5^ according to the percentage of hepatic fatty infiltration (0, <5%; 1, 5-33%; 2, >33-66%; 3, >66%). This criterion, unlike the one initially proposed by^1^, considers a minimum of 5% steatosis for the minimum operative definition of histological NAFLD in biopsy specimens from adults and children. For statistical analysis of each QUS parameter, animals in the study were divided according to that classification into grade 0 (N=24), grade 1 (N=15) and grade 2 (N=16) of steatosis. Representative liver tissue sections are shown in Fig 2, which revealed a considerable extent of macrovesicular steatosis in the diet group. The mean fat fraction percentage of grade 0 subjects was $1.28 \pm 1.26$  for grade 1 was $20.36 \pm 7.36$ and for grade 2 was $45.32 \pm 9.74$ (*p*-value < 0.01).

**Figure 3:**
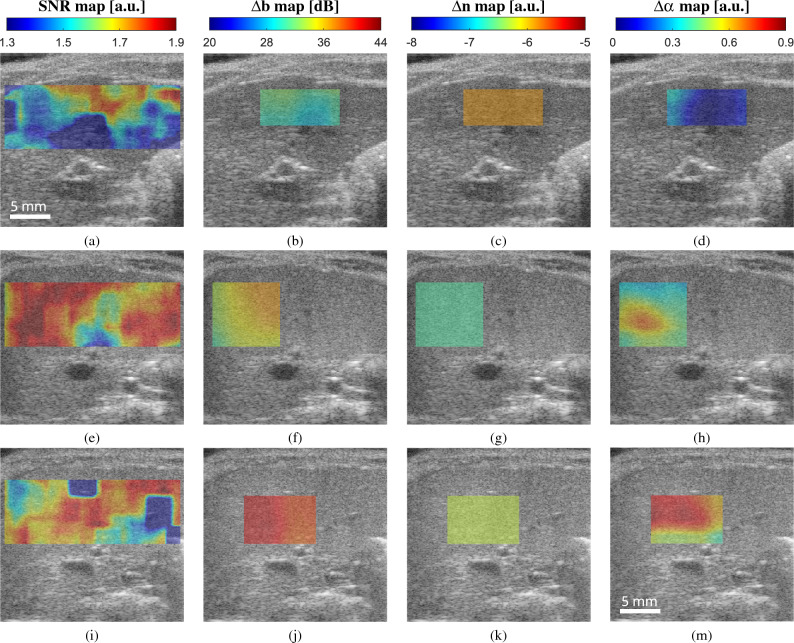
SNR and estimated coefficients ($\Delta \text {b}$, $\Delta \alpha$, $\Delta \text {n}$) maps for a particular subject in the Fatty group with: (**a**)–(**d**) Grade 0; (**e**)–(**h**) Grade 1 and (i)-(m) Grade 2.

**Figure 4:**
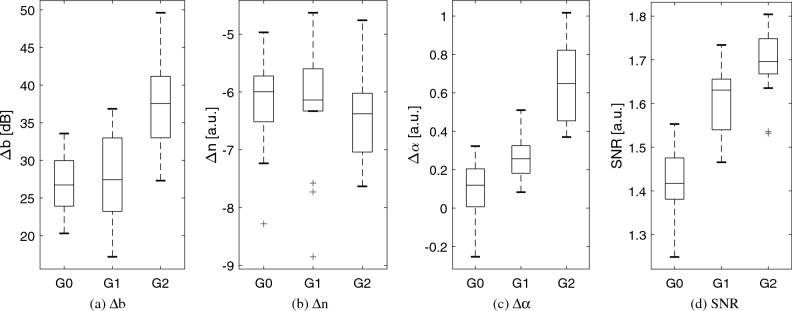
Box plots of estimated coefficient maps $\Delta \text {b}$, $\Delta \alpha$, $\Delta \text {n}$ and SNR maps median values for grades 0 (G0), 1 (G1) and 2 (G2).

**Figure 5:**
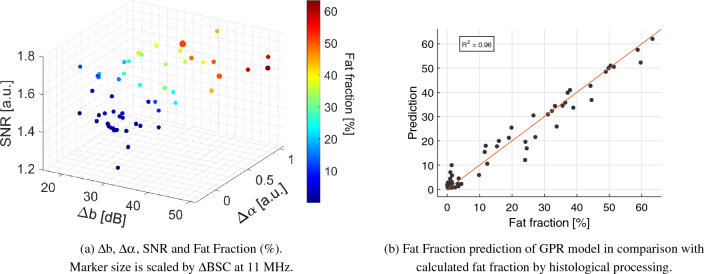
QUS coefficients three-dimensional distribution and GPR model results.

Computation of SNR was added as a metric of homogeneous texture^48^ to quantitatively identify a region of interest (ROI) with low presence of heterogeneities, and, mainly, to differentiate those relative AC and BSC values that the algorithm cannot estimate properly due to tissue irregularities. The SNR map was computed in a fixed area that fully encompass the liver parenchyma, then, the ROI for RPL-TV algorithm computation was chosen where a nearly uniform spectral pattern^49^ (SNR close to 1.91) is found, in a subsection of the aforementioned fixed area. Estimated coefficient maps computed by RPL-TV algorithm and SNR map calculation are shown in Fig. 3 for each grade of steatosis on our dataset. However, not all irregularities in the liver can be avoided, especially in control group rats (grade 0 steatosis) which were found to have a higher amount of heterogeneities in the B-mode US images. Because of their low fat fraction percentage, more vascularities and other tissue components are visible in the liver parenchyma. These intrahepatic structures are blurred with steatosis progression in B-mode images^50^. For MCD diet fed rats, we observed that both relative AC and BSC estimates increased while steatosis grade also increased following previously reported tendencies^30^. However, for a group of grade 0 rats with lower SNR, relative AC and BSC values were higher than expected, which can be related to a reduction in the performance of the estimation algorithm when applied to a ROI with high degree of heterogeneity. On the other hand, on rats with higher SNR values we can differentiate between grades 1 and 2 of liver steatosis. Moreover, this trend can also be observed when analysing median values as shown in Fig. 4. Even if there are no discernible differences between each grade for every parameter, the overall contribution of $\Delta \alpha$, $\Delta \text {b}$ $\left( \text {dB}\right)$, $\Delta \text {BSC}$ at 11 MHz, and SNR is significant as it is reported in *p*-values obtained by Kruskal-Wallis Test in Table 1. Fig. 5 A shows a three-dimensional distribution of QUS coefficients $\Delta \text {b}$, $\Delta \alpha$, and SNR in which the marker size is relative to $\Delta$ BSC at 11 MHz and colormap is given by fat fraction percentage. From this plot, it is clear that animals with low fat fraction percentage are related with a lower value of SNR.

**Figure 6:**
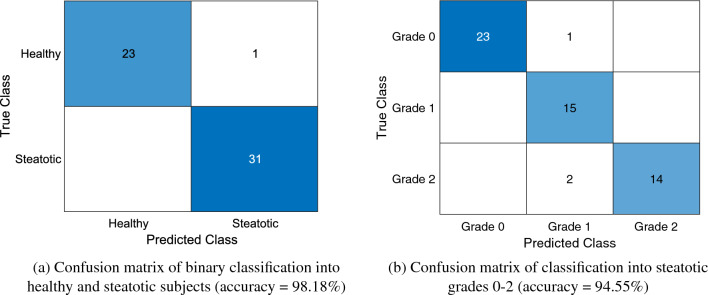
Classification results of GPR model.

A gaussian process regression (GPR) model was trained using as inputs the median values of $\Delta \text {b}$, $\Delta \alpha$, $\Delta \text {BSC}$ at 11 MHz and SNR. The model used an exponential kernel with no basis function. Moreover, leave-one-out (LOO) cross-validation was used to increase the robustness of the model. In addition, as fat fraction percentage should be positive, regression model predictions were passed through a ReLU function. After that, the model achieved an $R^2$ value of 0.97, a Root Mean Square Error (RMSE) of 3.64, and significant *p*-values (<0.01), for all variables used for training. Based on estimated fat fraction percentage, on test predictions, the model reached an accuracy of 98.18% to differentiate between healthy (grade 0) and steatotic groups (grades 1-2). Then, when classifying samples into the different steatosis grades 0-2, an overall accuracy of 94.55% was achieved. Confusion matrices are shown in Fig. 6. Regarding training results, classification accuracy values were 99.97% and 99.90%, for binary and multiple classification, respectively. These values were obtained as an average of prediction results on every model trained during LOO validation.

**Table 1:** Median (IQR) of $\Delta \alpha$ $\left( \text {a.u.}\right)$, $\Delta \text {b}$ $\left( \text {dB}\right)$, $\Delta \text {n}$ $\left( \text {a.u.}\right)$, $\Delta \text {BSC}$ at 11 MHz $\left( \text {a.u.}\right)$, $\text {SNR}$ $\left( \text {a.u.}\right)$ and mean±std Fat Fraction $\left( \text {\%}\right)$ with their *p*-value (Kruskal-Wallis Test). .

**Grade**	$\Delta \text {b}$	$\Delta \text {n}$	$\Delta \alpha$	SNR	$\Delta \text {BSC}\times 10^{-4}$	Fat Fraction
0	26.72(6.05)	$-6.00(0.79)$	0.12(0.20)	1.42(0.09)	2.35(5.42)	$1.28\pm 1.26$
1	27.41(9.76)	$-6.14(0.73)$	0.26(0.14)	1.63(0.12)	1.60(7.83)	$20.36\pm 7.36$
2	37.55(8.18)	$-6.38(1.02)$	0.65(0.37)	1.70(0.08)	41.95(48.62)	$45.23\pm 9.74$
*p*-value	$1.65\times 10^{-5}$	0.46	$1.02\times 10^{-8}$	$5.91\times 10^{-9}$	$2.39\times 10^{-3}$	$5.91\times 10^{-11}$

## Discussion

Our analysis was performed using a murine animal model, for that reason, higher frequencies were selected when imaging the liver which could increase the risk of poor signal-to-noise conditions. However, as showed in^51^, the correlation between fat fraction and BSC is significant in all frequencies below 30 MHz. Moreover, the computation results were improved by averaging the median values of estimated QUS coefficients over a sequence of 20 RF data frames. Furthermore, this study demonstrates that RPL-TV algorithm allowed fat fraction percentage estimation more accurately on steatotic subjects (Fig. 5), this performance could be caused due to the reduction of intrahepatic blood vessels in liver parenchyma which results into a more homogeneous texture in the ROI^22^.

By analyzing the distribution of samples, higher fat fraction results into an increase in QUS-based relative AC and BSC parameters. When comparing steatosis grades 0 (1.28% of fat fraction) and 2 (45.23% of fat fraction), the relative value of AC and BSC increased by approximately 0.40 and 10 times, respectively. However, the parameter that contributes significantly to found a higher BSC in fatty liver is $\Delta \text {b}$ (*p*-value < 0.01), while $\Delta \text {n}$ does not exhibit a particular trend with the increase of steatosis. It has been shown that main sources of acoustic scatters in the liver are fat droplets and hepatocyte nuclei^51^. Liver steatosis causes an increase in fat droplet deposition in hepatocytes which alters the locations of the hepatocyte nuclei within the hepatocytes, changing the spatial distribution of the nuclei and increasing BSC^51^. When the scattering source is small compared to the wavelength, scattering sources act as Rayleigh scatterers^52^ showing a frequency dependence of $f^4$. In this study, all analyzed structures (mainly nuclei and fat droplets) are smaller than the used wavelength, as a reference, fat liver droplets reach a diameter of approximately 20$\mu$m and the wavelength was 140$\mu$m. Thus, scattering should be consistent with a Rayleigh behavior and proportional to $f^4$, so it is expected for $\Delta \text {n}$, representing frequency dependence of BSC, to be close to a constant. In addition, AC and BSC differences between healthy and fatty livers are consistent with those reviewed previously in literature^30^ where the AC increased 0.56 dB/cm/Hz when lipid content in the liver was 9 times higher. Moreover, in relation with relative BSC, it is in the same order of magnitude of values reported in literature where BSC increased 10 times^30,36^.

In addition, B-mode images were affected by an increased brightness ($\Delta \text {b}$) caused by intracellular accumulation of fat vacuoles^53^, matching common qualitative visual assessment of echogenicity for hepatic steatosis. Besides, a change in the distribution of hepatocyte nuclei occurs as a consequence of emerging fat droplets, leading to a change in the structure function which contributes to the positive correlation between fat fraction and BSC^51^. Furthermore, the measurement of inhomogeneity in tissue given by SNR was one of the most significant parameters as it allows to differentiate mainly between control and diet groups of rats (Fig. 4 D). When fat fraction increases, SNR median increases, approaching to the average SNR of Rayleigh distribution (1.91) that implies homogeneity^49^, as intrahepatic structures presence in the steatotic tissue are reduced. The classification performed with regression results showed that proposed method is capable of staging liver steatosis. One limitation of this study is that it does not include grade 3 cases of hepatic steatosis. However, it is known that higher grades of steatosis are associated with increased AC and BSC values^29^. Moreover, the kernel function used in GPR allows the model to capture complex non-linear trends in the underlying structure of the data^54^. Therefore, it is likely that the trained GPR model will capture the increasing trend in the input-output relationship and predict higher fat fraction estimates for grade 3 cases. In addition, as it employs the instrumentation used for conventional US, its introduction to clinical setting has reduced equipment limitations^55^.

Finally, our QUS approach demonstrates that QUS coefficients estimates obtained by the adapted RPL-based algorithm together with SNR have the potential for assessing the progression of NAFLD and non-invasive quantification of liver fat content.

## Methods

### Animal Model

All procedures were carried out in accordance with relevant guidelines and regulations. Animal experiments were performed based on a protocol approved by the Institutional Animal Care and Use Committee (IACUC) at the University of Texas at Dallas. This manuscript complies with the ARRIVE guidelines for reporting animal research^47^. Ultrasound imaging measurements were performed on a group of 55 12-week-old Sprague-Dawley rats divided into control and diet groups^56,57^. To obtain different steatosis grades, imaging was performed either at 2 or 6 weeks. Control animals were fed standard chow, whereas the MCD group received a special diet (MP Biomedicals, Solon, OH). Animals were kept under a 12-h day/night rhythm with free access to food and water. After imaging session at week 2 or 6, all animals were humanely euthanized by performing a bilateral pneumothorax and cardiac puncture under isoflurane anesthesia, then, their livers were excised for histological processing. The dataset was divided into grade 0 (N=24), grade 1 (N=15) and grade 2 (N=16) of steatosis.

### Liver imaging protocol

Animals were anesthetized with 1–2% isofurane in oxygen (V3000PK, Parkland Scientifc, Coral Springs, FL) and placed on a temperature-controlled heating pad to maintain core levels (Rodent Surgical Monitor, AnimaLab, Poznan, Poland). For each animal, the US transducer was positioned and fixed in the transverse plane after co-visualization of major blood vessels, including the aorta, inferior vena cava (IVC), and portal vein. Reconstructed RF data was collected using the VEVO-3100 scanner (FUJIFILM VisualSonics Inc, Toronto, Canada) equipped with an MX201 linear array transducer. Quadrature data was acquired at center frequency of 15 MHz, then it was converted to RF data, the process consists of an up sampling by factor of 8. The envelope of the original unfiltered data was used to generate the B-mode US images which have a lateral and axial distance of 23 mm and 21.7 mm, respectively. After the last imaging session for each dataset, animals were euthanized and livers excised. The right medial and left lateral lobes of the liver ($\ge$ 50% of each lobe) were fixed in 10% neutral-bufered formalin for at least 7 days at room temperature. Liver tissue was then embedded in paraffin, sectioned (5 $\mu m$), and mounted. H &E stains were used for morphological analyses. Digital images were acquired with a microscope at a magnifcation of 100$\times$ (Axio Scope.A1, Carl Zeiss, Tornwood, NY). To minimize the effect of sampling error, more than three histological images from right medial and left lateral lobes of the liver were used to calculate fat fraction percentage^18^. Quantification of fat fraction percentage was performed using a custom MATLAB software (MathWorks Inc, Natick, MA). This algorithm developed for fat vacuole segmentation consisted of the following steps: (1) 8-bit RGB digital images were converted to grayscale using *rgb2gray* function, (2) 8-bit grayscale images were binarized applying a simple global threshold which was manually selected for each image and had an approximate value of 180, (3) morphological operations of erosion and dilation were applied using a non-flat, ball-shaped structuring element with a radius and a maximum offset height of 5 implemented with *imerode*, *imdilate* and *offsetstrel* functions, (4) identification of connected elements was performed using *bwconncomp* function with a connectivity of 8, (5) intrahepatic structures other than vacuoles were manually chosen and removed, (6) fat infiltration percentage was determined by calculating the area occupied by the fat droplets as a fraction of the total area. In step 5, fat droplets were visually differentiated from other empty spaces based on their distinctive roundish shape, size and adjacency (in the case of clustered droplets) in comparison with vessels, sinusoids, bile ducts, and tissue cracks^58,59^. Structures such as nuclei, cytoplasm, and red blood cells were removed in step 2.

### Estimation framework

RF data processing was performed in a bandwidth of 4-18 MHz over a volume of 20 frames. This bandwidth was set to encompass frequency information data from the entire group of animals, above a noise power level of about -20dB. On each frame, SNR was obtained in a region that fully encompass the liver parenchyma, which had a fixed lateral and axial distance of 22 mm and 8 mm, respectively. Then, the ROI for RPL-TV algorithm was chosen in a subregion with an SNR close to 1.91 looking to reduce the amount of irregularities and vascularities that could bias the results, most of these artifacts can also be identified in B-mode images. To implement RPL-TV algorithm, the hyperparameters used in a previous study^46^ served as a reference, then they were manually tuned for this dataset which includes a greater diversity of steatosis degrees. The objective was to find a balance between estimation noise reduction and over-regularization. Here, RPL-TV algorithm was performed with the fixed hyperparameters $\mu _b = 10^0$, $\mu _n = 10^3$ and $\mu _a = 10^3$. The power spectrum was calculated using $20\lambda \times 20 \lambda$ ($\lambda$ = 0.14mm) data blocks with and 80% of overlap, each data block passed through a Tukey Window with a ratio of 0.25. The continuous model is obtained by comparing the spectra in the sample *s* and the reference *r*. Moreover, backscattering and total attenuation are modelled as in^44^. Then, dividing the spectra and taking logarithm, we obtain

$$\begin{aligned} \log \left[ \frac{S_s(f,z)}{S_r(f,z)}\left( f\right) T_m\left( f\right) \right] = \log \left( \frac{b_s}{b_r}\right) + \left( n_s-n_r\right) \cdot \log \left( f\right) - 4\int \left( \alpha _s(z)-\alpha _r(z)\right) f\text {d}z, \end{aligned}$$

where $T_{m}$ is the transmission compensation function of phantom’s protective membrane, *b* is the BSC amplitude, *n* represents the BSC frequency dependence and $\alpha$ is the effective attenuation coefficient. Then, by denoting $Y(f,z) = \log \left[ \frac{S_s(f,z)}{S_r(f,z)}\left( f\right) T_{m}\left( f\right) \right]$, estimated coefficient maps are defined as $\Delta \text {b}\left( z\right) =\frac{b_{s}}{b_{r}}$, $\Delta \text {n}\left( z\right) =n_{s}-n_{r}$ and $\Delta \alpha \left( z\right) =\alpha _{s}\left( z\right) -\alpha _{r}\left( z\right) =\frac{d}{dz}\Delta \text {a}\left( z\right)$. Therefore, the adapted continuous model is

1 $$\begin{aligned} Y\left( f,z\right) = \log \left( \Delta \text {b}\left( z\right) \right) +\Delta \text {n}\left( z\right) \cdot \log {\left( f\right) }-4f\cdot \Delta \text {a}\left( z\right) , \end{aligned}$$

Finally, relative backscatter coefficient is defined as (2):

2 $$\begin{aligned} \Delta \text {BSC}(f) = \Delta \text {b} \cdot f^{\Delta \text {n}}, \end{aligned}$$

The envelope SNR was defined as in^48^,

3 $$\begin{aligned} \text {SNR}=\frac{\langle \textrm{E}\rangle }{\sqrt{\left\langle (\textrm{E}-\langle \textrm{E}\rangle )^2\right\rangle }} \end{aligned}$$

where $\langle \cdot \rangle$ denotes expectation, and $\textrm{E}$ represents the RF signal envelope values. SNR was computed using $20\lambda \times 20 \lambda$ data block to obtain a two-dimensional map.

### Regression model

The GPR model is a bayesian nonparametric probabilistic model based on kernel used for supervised learning which aims to reconstruct the underlying signal by removing the noise^54^. It has several advantages for operating in complex and small datasets with non-linear distribution thanks to its ability to provide measures of uncertainty, interpolation of predictions and high flexibility to adjust to non-linear distributions^54^. This model was trained using an exponential kernel function with no basis function. Data standardization, LOO cross-validation and hyperparameter optimization of kernel parameters were performed. As LOO was implemented, the training set consists of 54 samples and test set of 1 sample randomly selected for each of the 55 folds. Moreover, the regression model inputs were selected based on the significance of each parameter obtained during statistical analysis. Classification groups were divided into grades of liver steatosis based on fat fraction percentage obtained by histological processing.

### Statistical analysis

The results of QUS parameters for each subject were summarized as median(IQR). Additionally, taking in to account that the data did not follow a normal distribution, a nonparametric method was used to evaluate the significance of each parameter individually. This method was the Kruskal-Wallis^60^ test that was performed based on groups divided by steatosis grades 0, 1 and 2. For this purpose, a *p*-value of less than 0.01 was considered statistically significant. All statistical analyses were performed with *Statistics and Machine Learning Toolbox* of MATLAB R2021a.

## Data Availability

The datasets used and/or analysed during the current study available from the corresponding author on reasonable request.
